# Post-Transcriptional Regulation of Transforming Growth Factor Beta-1 by MicroRNA-744

**DOI:** 10.1371/journal.pone.0025044

**Published:** 2011-10-04

**Authors:** John Martin, Robert H. Jenkins, Rasha Bennagi, Aleksandra Krupa, Aled O. Phillips, Timothy Bowen, Donald J. Fraser

**Affiliations:** School of Medicine, Institute of Nephrology, Cardiff University, Cardiff, Wales, United Kingdom; University Medical Center Freiburg, Germany

## Abstract

Transforming Growth Factor Beta-1 (TGF-β1) is a pleiotropic cytokine that is of central importance in wound healing, inflammation, and in key pathological processes including cancer and progressive tissue fibrosis. TGF-β1 is post-transcriptionally regulated, but the underlying mechanisms remain incompletely defined. Previously, we have extensively delineated post-transcriptional regulation of TGF-β1 synthesis in the kidney, with evidence for relief of translational repression in proximal tubular cells in the context of diabetic nephropathy. In this study, we have investigated the role of the TGF-β1 3′Untranslated Region (3′UTR). Two different 3′UTR lengths have been reported for TGF-β1, of 543 and 137 nucleotides. Absolute quantification showed that, while both UTR lengths were detectable in various human cell types and in a broad range of tissues, the short form predominated in the kidney and elsewhere. Expression of both forms was up-regulated following auto-induction by TGF-β1, but the short:long UTR ratio remained constant. Incorporation of the short UTR into a luciferase reporter vector significantly reduced reporter protein synthesis without major effect on RNA amount, suggesting post-transcriptional inhibition. *In silico* approaches identified multiple binding sites for miR-744 located in the proximal TGF-β1 3′UTR. A screen in RNA from human tissues showed widespread miR-744 expression. miR-744 transfection inhibited endogenous TGF-β1 synthesis, while direct targeting of TGF-β1 was shown in separate experiments, in which miR-744 decreased TGF-β1 3′UTR reporter activity. This work identifies miR-744-directed post-transcriptional regulation of TGF-β1 which, given the pleiotropic nature of cellular responses to TGF-β1, is potentially widely significant.

## Introduction

Transforming growth factor-β_1_ (TGF-β_1_) directs cellular responses including proliferation, differentiation, migration, and survival. TGF-β_1_ is a key regulator of embryogenesis, angiogenesis, inflammation, and wound healing. Aberrant TGF-β_1_ synthesis is implicated in numerous pathological processes including tumorigenesis, atherosclerosis and fibrosis (reviewed in [1], [2]). Thus, understanding the regulation of TGF-β_1_ expression is of importance in homeostatic regulation and disease.

Disparities in TGF-β_1_ expression at the level of mRNA and protein suggest post-transcriptional regulation [3], [4]. Polysome analysis confirms that TGF-β_1_ mRNA is inherently poorly translated, *in vitro* and *in vivo* [5], [6]. Our previous work demonstrates specific activation of TGFβ translation in renal epithelial cells in response to stimuli including Glucose, Platelet Derived Growth Factor, and TGF-β_1_ itself [7], [8], [9]. Subsequently, we have studied the mechanisms by which TGF-β_1_ translation is regulated. The 5′ and 3′ Untranslated Regions (UTRs) are key *in cis* sites for post-transcriptional control. We have previously studied the 5′UTR of TGF-β_1_. We have identified an interaction between the 5′UTR and the RNA/DNA binding protein YB-1 [10] that regulates its translational activity, and have characterised a translation-inhibitory stem loop motif [11].

In the current study, we have examined the role of the TGF-β_1_ 3′UTR in post-transcriptional regulation, and the potential for regulation of TGF-β_1_ by microRNAs (miRs). miRs are small, endogenous, non-coding RNAs that inhibit gene expression post-transcriptionally, principally via interaction with target recognition sites in the 3′UTRs of regulated genes. Relevant for potential targeting by miRs, two distinct 3′UTR lengths have been reported for TGF-β_1_, length 543 and 137 nucleotides [12], [13]. This is in keeping with recent reports, which suggest that for a proportion of human genes 3′UTR length may vary dependent on alternate polyadenylation sites, selection of which may be regulated during development and in response to cellular cues [14] (and reviewed in [15]). Two potential polyadenylation signals are found within the TGF-β_1_ 3′UTR. The hexanucleotide AAUAAA is the predominant sequence directing cleavage and polyadenylation of pre-mRNA [16]. This sequence is found at position 498 following the stop codon of TGF-β_1_. Other less conserved AU- or A-rich sequences have been observed in the 3′ end of a smaller fraction of transcripts. One such sequence, AUUAAA, is present at position 110 of the TGFβ 3′UTR.

In this work we have investigated expression and function of the TGF-β_1_ 3′UTR variants. We show that 543-nucelotide and 137-nucleotide variants are expressed, and that both variants inhibit heterologous reporter gene expression, apparently predominantly via post-transcriptional mechanisms. Detailed expression studies show that the transcript containing a 543-nucelotide UTR is a minor component of TGF-β_1_ mRNA across a range of human tissues and cells, while the 137-nucleotide UTR predominates. Subsequently, we have identified miR-744 as targeting the 137-nucleotide UTR, identifying a microRNA-mediated mechanism of post-transcriptional regulation of TGF-β_1_.

## Results

### Detection and Characterisation of the TGFβ_1_ 3′UTR

**Figure 1:**
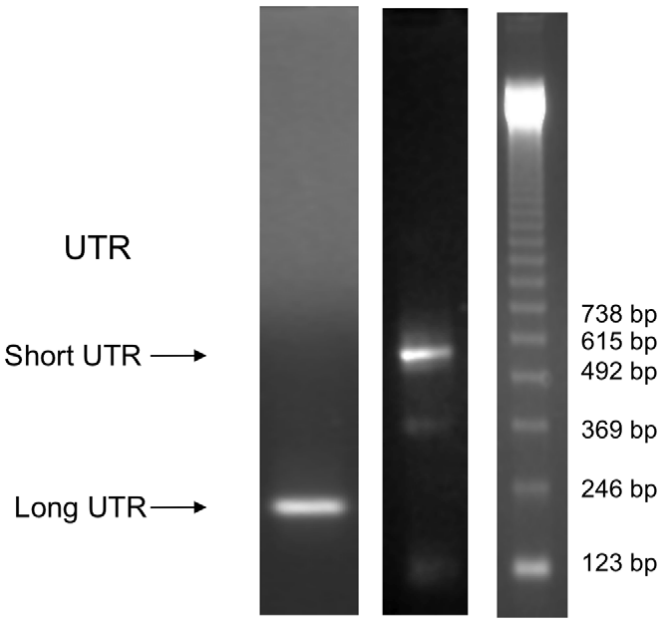
RT-PCR detection of short and long variants of TGF-β_1_ 3′UTR in HK-2 cells. Arrows indicate detected products of predicted size (137 and 543 nucleotides). The far right lane is a standard DNA ladder. N = 6.

**Figure 2:**
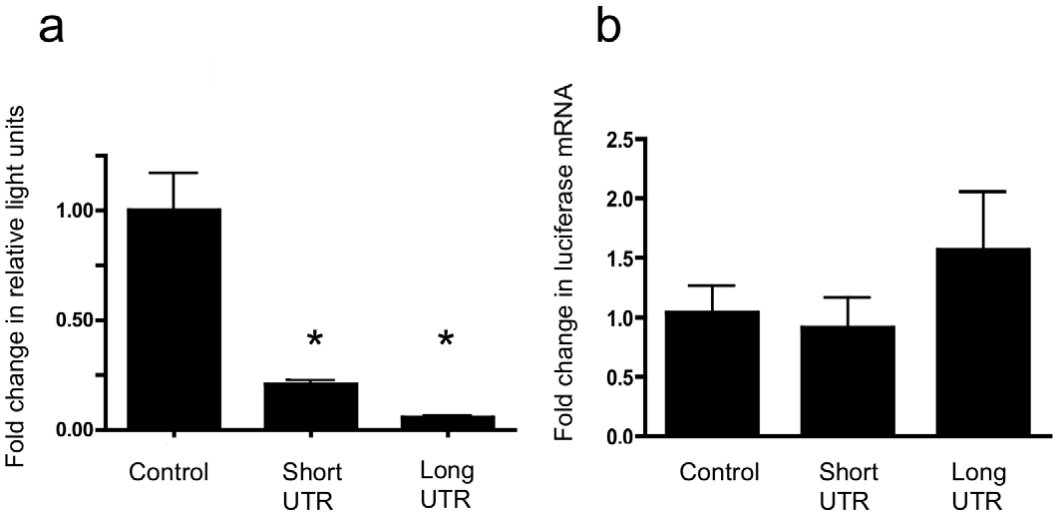
Effect of long and short TGF-β_1_ 3′UTRs on reporter gene expression. (a) Amount of firefly luciferase activity corrected for renilla activity of cells following transfection with pGL3 control empty vector, pGL3short and pGL3long. (n = 11) *p<0.0001 (b) Relative expression of firefly luciferase RNA detected by qPCR in cells following transfection with pGL3 control empty vector, pGL3short and pGL3long (p = NS).

We have previously demonstrated post-transcriptional regulation of TGF-β_1_ expression, and extensively characterised underlying mechanisms, using E6/E7 transformed proximal tubular epithelial cells (HK-2 cells). RT PCR showed expression of both long and short TGF-β_1_ 3′UTR variants in HK-2 cells (Fig. 1). Subsequently, reporter vectors were generated from the pGL3 plasmid incorporating the TGF-β_1_ 137 nucleotide (pGL3short) and 543 nucleotide (pGL3long) UTRs in an appropriate 3′ context downstream of the firefly luciferase open reading frame. Both TGF-β_1_ UTR vectors showed diminished reporter activity compared with control (Fig. 2a, pGL3short 79% reduction in control activity, pGL3long 94% reduction in control activity, p<0.0001 pGL3short, p<0.0001 pGL3long, compared to control vector). Quantification of luciferase RNA showed no significant differences between UTR vectors and control (Fig. 2b) suggesting that the effect of the UTR was predominantly at the level of protein synthesis rather than RNA stability.

### The predominant TGF-β_1_ isoform contains the 137-nucleotide UTR

**Figure 3:**
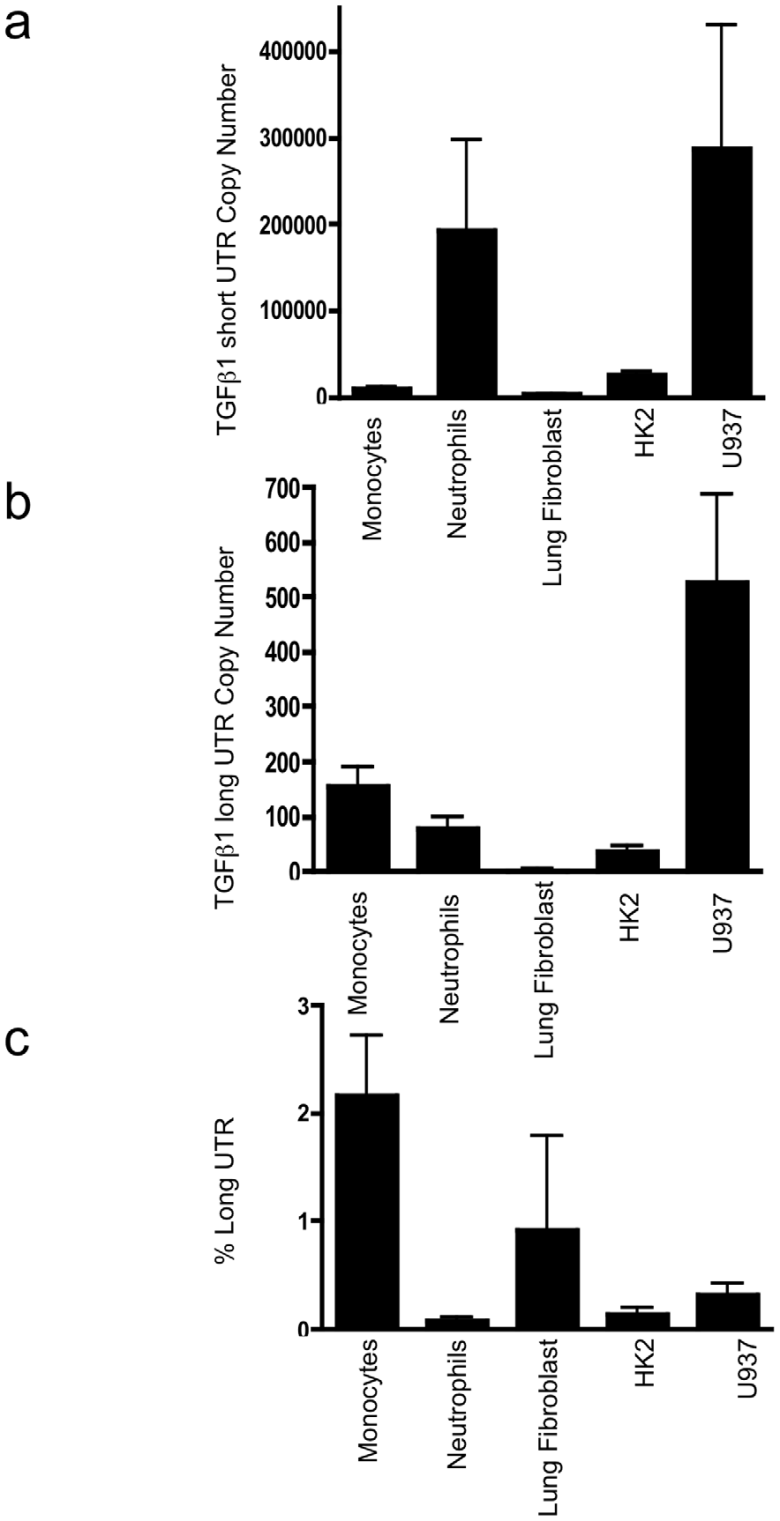
Copy number evaluation of short and long variants of TGF-β_1_ 3′UTR. The copy number of (a) short and (b) long forms of TGF-β_1_ 3′UTR detected in peripheral blood mononuclear cells (PBMC), polymorphonuclear cells (PMN), lung fibroblasts, HK2 (proximal tubular epithelial cells) and the monocyte cell line U937. (c) The percentage of total TGF-β_1_ mRNA with long UTR variant. (Data is from 3 independent experiments).

**Figure 4:**
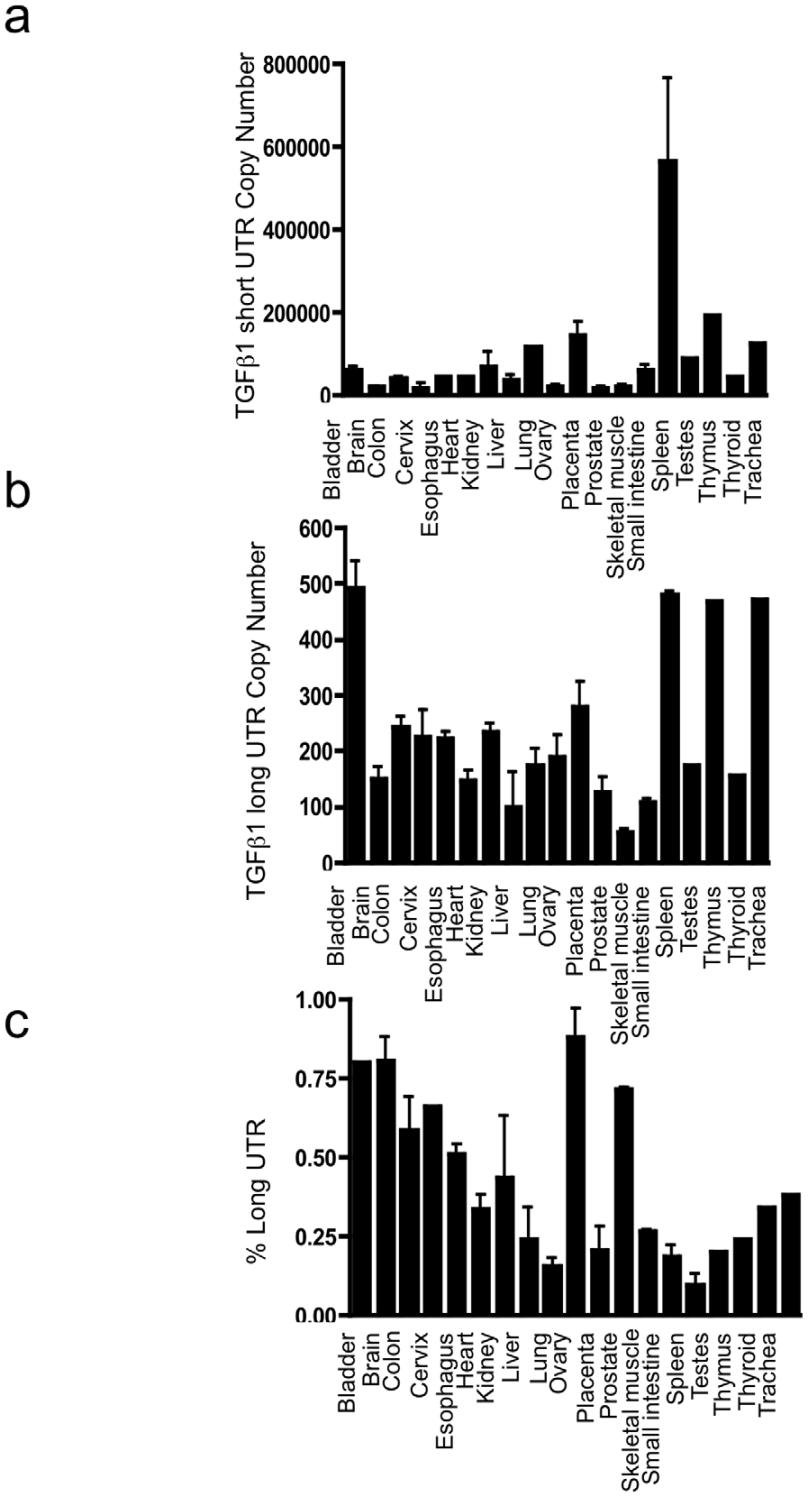
Copy number evaluation of short and long variants of TGF-β_1_ 3′UTR. The copy number of (a) short and (b) long forms of TGF-β_1_ 3′UTR present in 0.04 µg total RNA from 19 human tissues. (c) The percentage of total TGF-β_1_ mRNA with long UTR variant.

**Figure 5:**
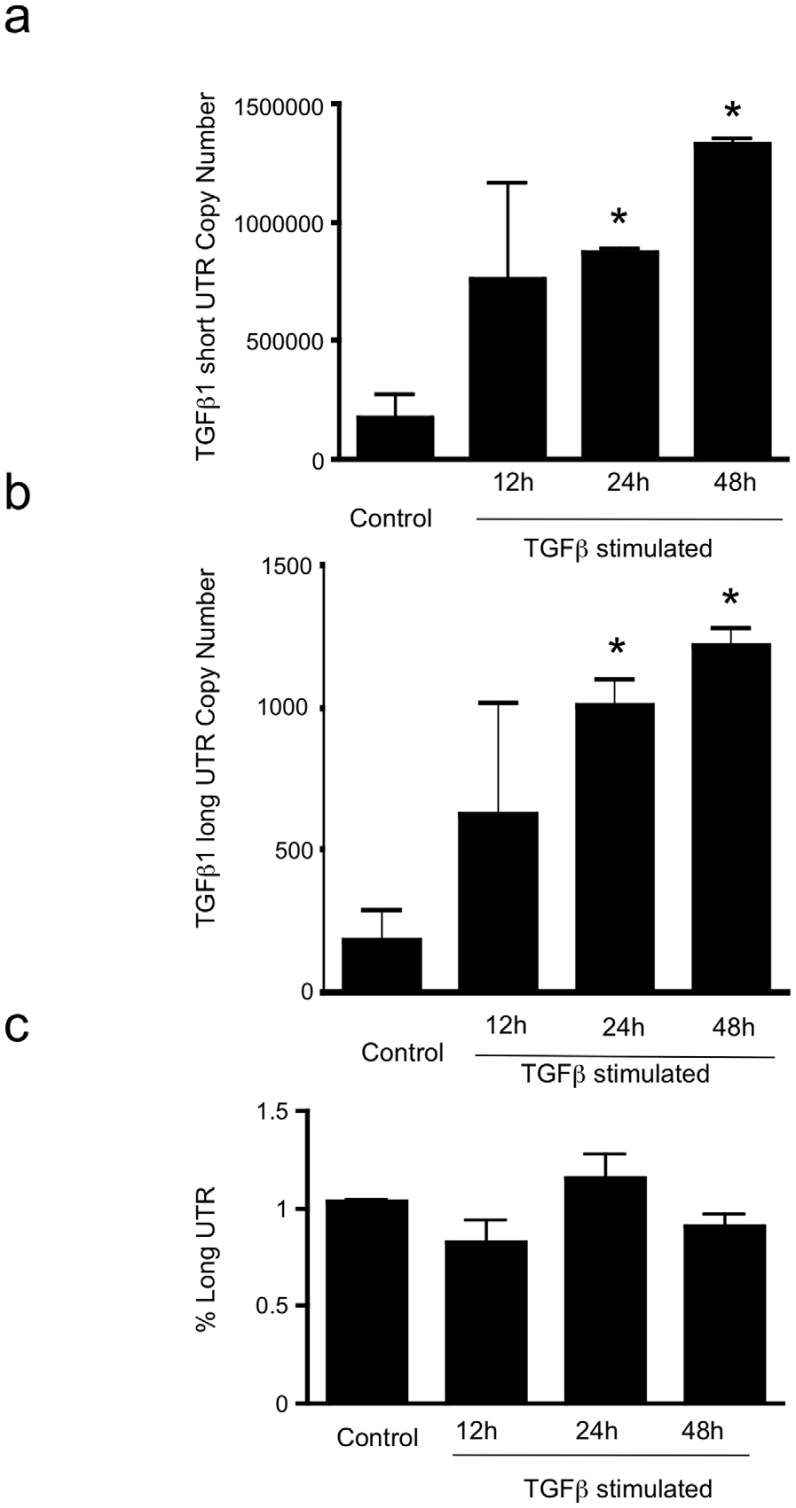
Effect of TGF-β_1_ autoinduction on long and short UTR variant expression. The copy number of (a) short and (b) long forms of TGF-β_1_ 3′UTR present (in 0.04 µg total RNA), and (c) The percentage of total TGF-β_1_ mRNA with long UTR variant present in unstimulated and TGF-β_1_ (10 ng/ml) stimulated HK2 cells. (n = 3) *p<0.005).

**Figure 6:**
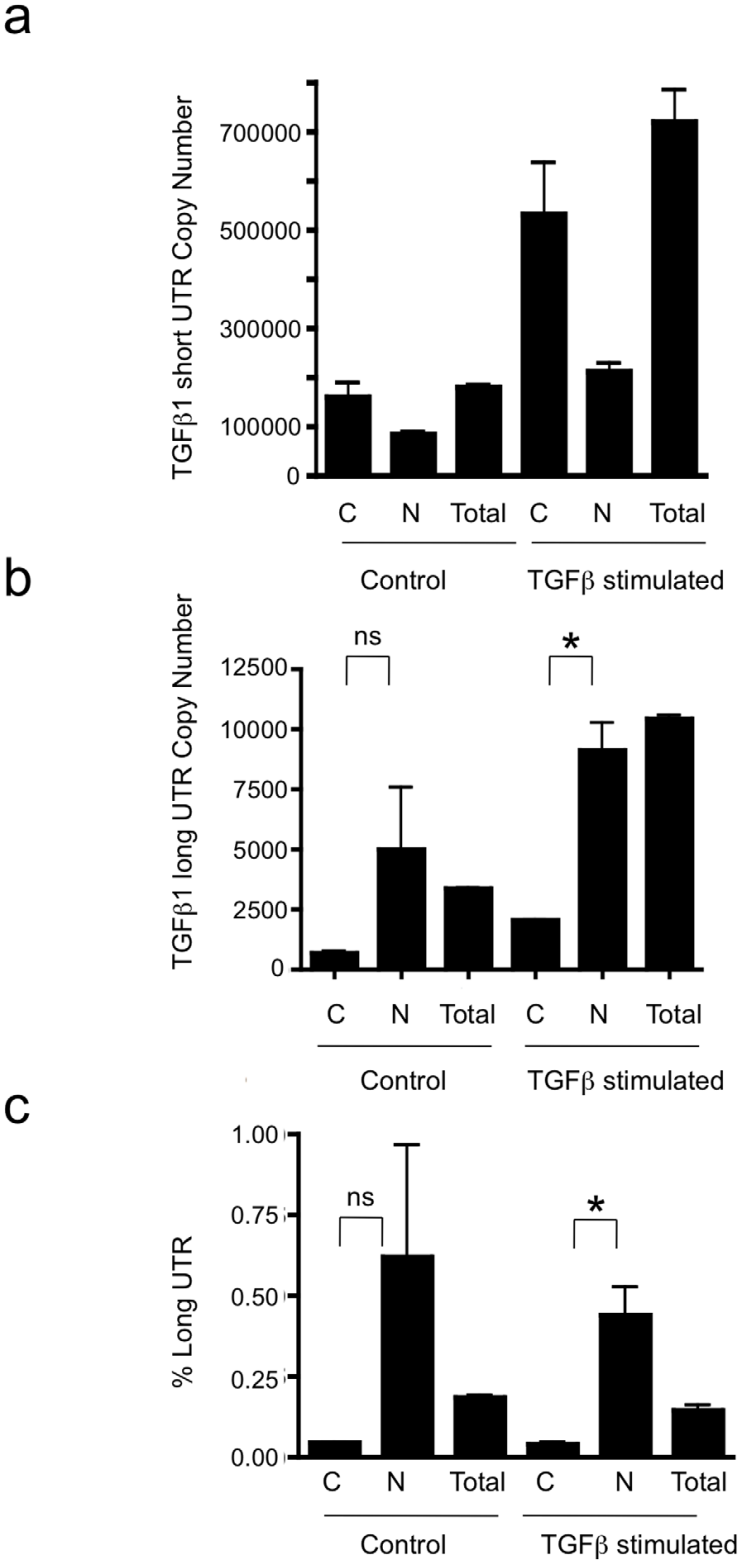
Nuclear vs. cytoplasmic copy number of long and short UTR variants. The copy number of (a) short and (b) long forms of TGF-β_1_ 3′UTR present (in 0.04 µg total RNA), and c) Long variant expression as a percentage of the total amount present in the cytoplasm, nucleus and total cell extract of unstimulated and TGF-β_1_ stimulated HK2 cells. (*p<0.05).

Subsequently, absolute quantification of long and short UTR variants was performed, utilising qRT PCR and standard curves of known copy numbers of plasmid-derived reference standard. Copy number evaluation was performed in cell lines of epithelial and mesenchymal lineage, together with monocytes and polymorphonuclear cells (Fig. 3 a: Short copy number, b: Long copy number, c: percentage of total transcript number that contains the long form UTR). Long and short UTR variants were detectable in all cell lines tested, however the long UTR variant was ubiquitously found at low copy number, typically representing 2% of total TGF-β_1_ mRNA. Similarly, a screen of 19 human tissues showed presence of the long UTR variant at low copy number (Fig. 4 a: Short copy number, b: Long copy number, c: percentage of total transcript number that contains the long form UTR). Subsequently we investigated whether increased TGF-β_1_ synthesis was associated with a change in the ratio of short to long TGF-β_1_ 3′UTR expression. TGFβ auto-induction is a well-recognised phenomenon. We have previously documented transcriptional and post- transcriptional TGFβ autoinduction in HK2 cells [17]. Incubation of HK-2 cells with TGF-β_1_ 10 ng/ml for 24 h led to enhanced TGF-β_1_ expression, however the ratio of short to long UTR variant remained constant (Fig. 5 a: Short copy number, b: Long copy number, c: percentage of total transcript number that contains the long form UTR). Interestingly, absolute quantification of long vs. short UTR transcript number in nuclear and cytoplasmic RNA preparations showed increased expression of the long UTR variant in nuclear RNA (Fig. 6). This is suggestive that the long UTR variant may be retained or degraded in the nucleus to a greater extent than the short UTR variant. However, in nuclear RNA, the short UTR variant remained the predominant transcript type.

Taken together, this data shows that in a wide range of cells and tissues the preponderant form of TGF-β_1_mRNA is the short form, originating from polyadenylation directed by the non-canonical ATTAAA site at position 110 of the TGF-β_1_ 3′UTR, and that polyadenylation at the canonical AATAAA site at position 498 of the TGF-β_1_ 3′UTR makes only a minor contribution. The predominance of the short UTR transcript is suggestive that important post-transcriptional regulation will be constrained to the first 137 nucleotides of the TGF-β_1_ UTR.

### microRNA-744 is highly conserved, and predicted to target TGF-β_1_

**Figure 7:**
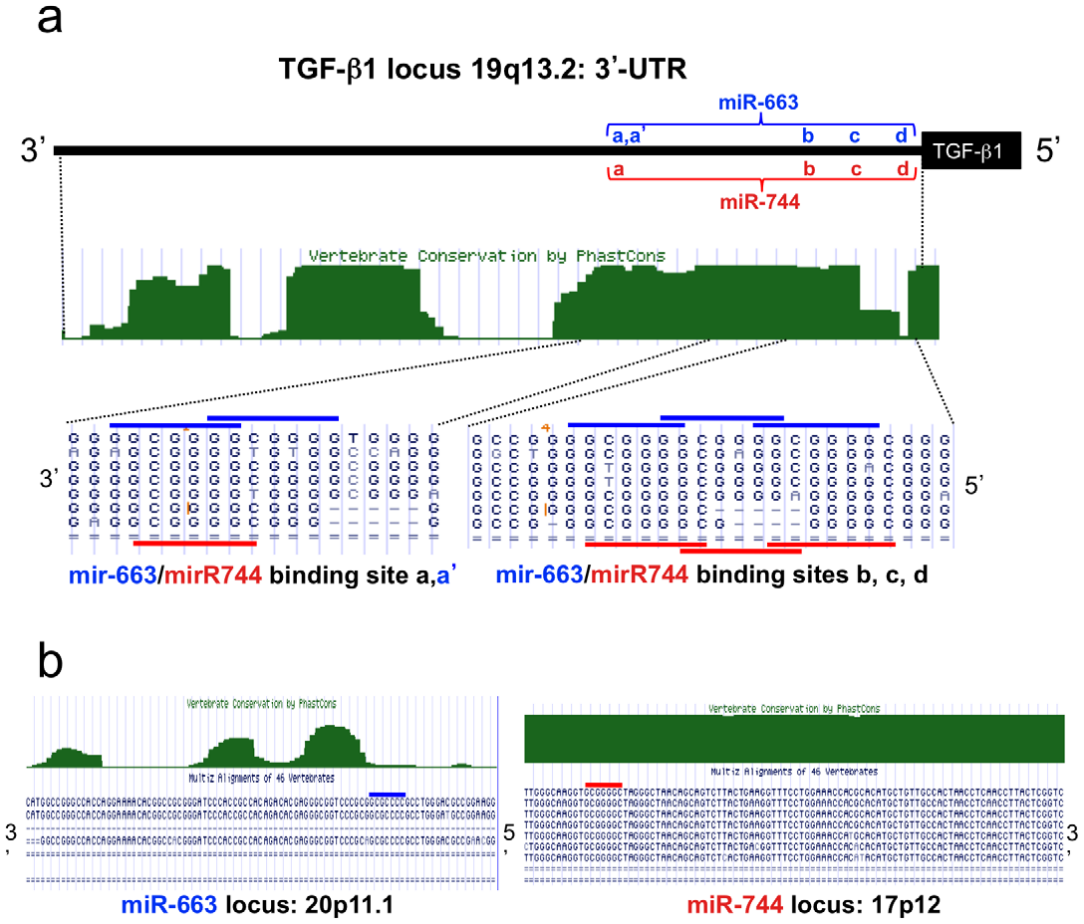
Vertebrate conservation and multi-species sequence alignments for the short form 3′-UTR of the TGF-β_1_ gene at 19q13.2 as well as miR-633 and miR-744 genomic loci. (a) multiple seed sequence recognition sites for miR-633 and miR-744 in the TGF-β_1_ 3′-UTR. The alignment features sequences from: hu, human; ch, chimpanzee; rm, rhesus macaque; ba, baboon, ma, marmoset; mo, mouse; el, elephant; xe, frog. (b) variable conservation across pre- miR-633 and pre-miR-744 sequences with alignment data from the same species. In each figure, seed sequences are highlighted by horizontal bars for miR-633 (grey) and miR-744 (black).

MicroRNAs are a recently described and generally important mechanism of post-transcriptional regulation of gene expression. MicroRNAs act principally *via* binding to sites in the 3′UTR of their targets. *In silico* prediction of microRNA/mRNA interactions relies on sequence complementarity, site homology with classical recognition sites, and conservation of sequence (reviewed in [18]). We used *in silico* approaches including Target Scan, Pictar and Miranda to predict potential microRNA regulation of TGF-β_1_. A total of 117 potential miR binding sites were identified in the TGF-β_1_ 3′UTR, of which 79 are distal to nucleotide 147, and are thus found only in the long UTR variant, while 38 are proximal to nucleotide 147, and so are common to both variants. In most cases, only a single binding site was identified for each miR. Multiple sites were identified for miR-663 and miR-744. Four binding sites for miR-744 were identified in close proximity in the proximal UTR, and interestingly an additional potential miR-744 site was identified in the open reading frame, between nucleotides 1875 and 1895. We have restricted the current analysis to sites in the 3′UTR, and have evaluated their evolutionary conservation, using UCSC Genome Browser (Fig. 7a). Three of the four miR-744 sites appear highly conserved in vertebrates, while the fourth site is identified only in H sapiens and simians (Fig. 7a). Interestingly a second miR, miR-663, shows considerable seed region homology with miR-744, and exhibits overlapping potential targeting sites within the TGF-β_1_ UTR (Fig. 7a). However, while miR-744 appears highly conserved in vertebrates, this is not the case for miR-663 (Fig. 7b). It is also notable that miR-663 is located in the heterochromatin close to the centromere of Chromosome 20, potentially limiting its expression in many contexts.

### The TGF-β_1_ 137-nucleotide UTR is a direct target of miR-744

**Figure 8:**
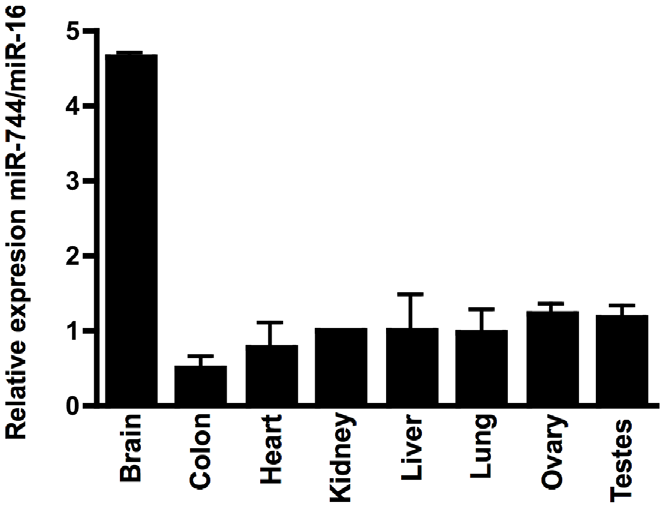
The expression of miR-744 in human tissues. The relative expression of miR-744 in RNA from 8 human tissues. Expression is calculated relative to miR-16, found to be uniformly expressed across all tissues, and normalized to kidney tissue expression. (Data is from three independent experiments).

**Figure 9:**
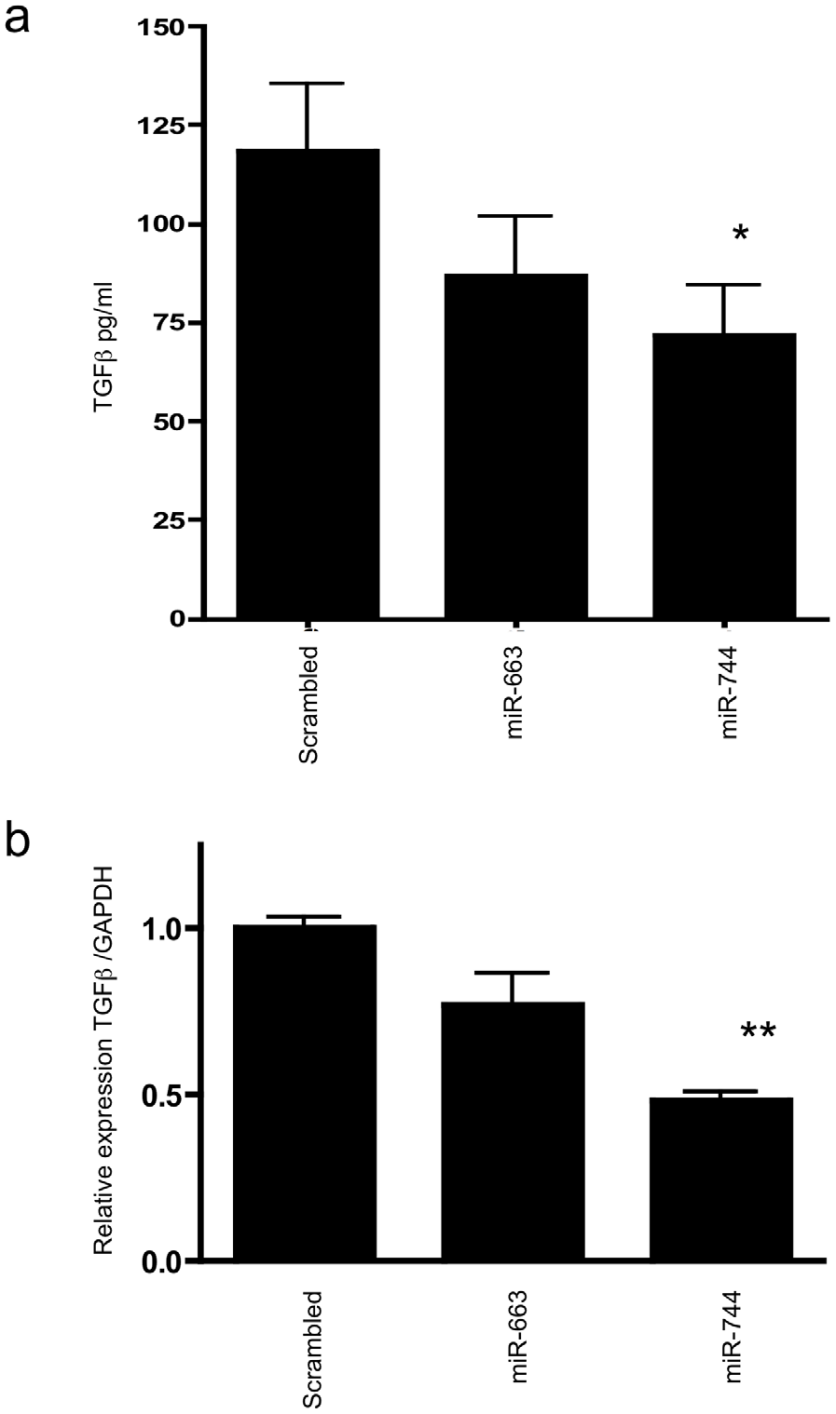
Effect of miR-663 and -744 transfection on endogenous TGF-β_1_ protein and message expression. (a) The amount of TGF-β_1_ protein detected by ELISA produced by cells 96 h following transfection with scrambled miR, miR-663 and miR-744. N = 3, *p<0.05. (b) The relative amount of TGF-β_1_ RNA detected by qPCR in cells following transfection with scrambled miR, miR-663 and miR-744. n = 3, **p<0.0001.

**Figure 10:**
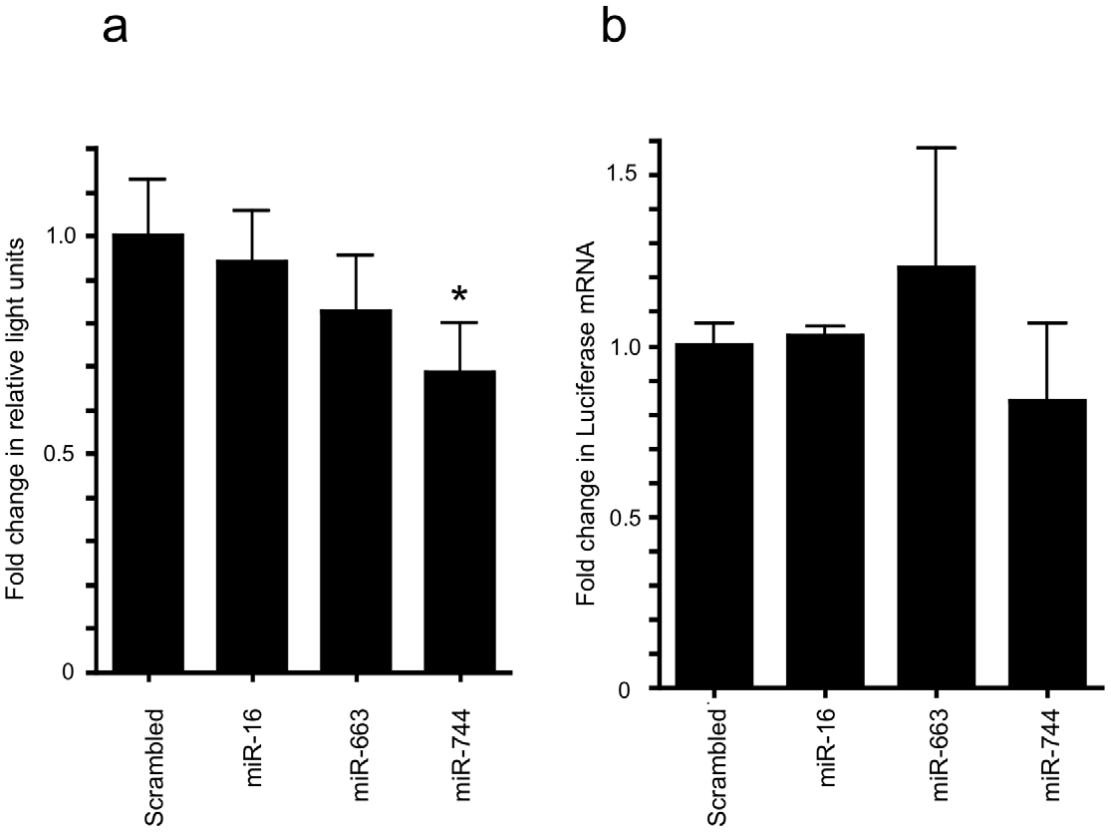
Effect of miR-663 and miR-744 transfection on TGF-β_1_ short UTR reporter vector. (a) The relative amount of luciferase activity corrected for renilla activity of cells following the co- transfection of pGL3short with scrambled miR, miR-16, miR-663 and miR-744. N = 10, *p<0.05. (b) The relative amount of luciferase RNA detected by qPCR in cells following the co- transfection of pGL3short with scrambled miR, miR-16, miR-663 and miR-744. N = 3 (p = NS).

We performed an initial screen in human tissues, and found widespread expression of miR-744 (Fig. 8). In order to test for microRNA-directed regulation of TGF-β_1_ synthesis, TGF-β_1_ was quantified in the cell culture supernatant of HK-2 cells transfected with miR-663 and -744 precursors. Transfection with miR-744 precursor led to a significant decrease in TGF-β_1_ release (Fig. 9a, control transfection 118.5pg/ml, miR-663 precursor transfection 87.0 pg/ml (p = n.s.), miR-744 precursor transfection 71.7 pg/ml (p<0.05)). Measurement of TGF-β_1_ mRNA by RT-qPCR also showed significant decreases in TGF-β_1_ mRNA in cells over-expressing miR-744 (Fig. 9b, compared to control transfection, there was 23% decrease with miR-663 precursor transfection (p = n.s.) and 52% decrease with miR-744 precursor transfection (p<0.0001)). Subsequently, the potential for direct targeting of the TGF-β_1_ 3′UTR by miR-744 was evaluated. Co-transfection of miR-744 precursor with pGL3short led to reduction in reporter vector activity (Fig. 10a, miR-663 precursor transfection, 15.8% reduction vs. control (p = n.s,) miR-744 precursor transfection, 34.0% reduction (p<0.05)). In contrast, transfection of miR-744 or of control miRs did not significantly alter luciferase mRNA generation (Fig. 10b). This contrasts with the reduction in endogenous TGF-β_1_ mRNA seen following miR-744 transfection (Fig. 9b). This may reflect inherent differences in TGF-β_1_ and luciferase reporter mRNA stability, or relate to the potentially higher luciferase mRNA copy number seen following transfection, compared to endogenous TGF-β_1_ mRNA copy number.

These data confirm that miR-744 directly targets the predominant, short, isoform of the TGF-β_1_ 3′UTR, and are suggestive that reduced miR-744 expression may be associated with increased TGF-β_1_ synthesis.

## Discussion

In this paper, we have analysed the structure and function of the TGF-β_1_ 3′Untranslated Region (3′UTR). TGF-β_1_ is extensively post-transcriptionally regulated, and we have previously demonstrated specific activation of TGF-β_1_ translation in response to stimuli including Glucose, Platelet Derived Growth Factor, and TGF-β_1_ itself [7], [8], [9]. Two lengths of TGF-β_1_ 3′UTR have been described [12], [19]. We have confirmed that both potential polyadenylation sites are utilised, leading to two alternate lengths of 3′UTR, but have found that the predominant form in all human cells and tissues studied is the 137 nucleotide “short” 3′UTR, derived from the alternate polyadenylation signal AUUAAA, rather than the consensus AAUAAA present at position 498.

The AAUAAA polyadenylation motif is very highly conserved in human cells and mutation in any of the nucleotides in this canonical sequence disrupts cleavage and polyadenylation of transcripts [16]. However, variant motifs occur, of which the AUUAAA motif present in the TGF-β_1_ 3′UTR is the most common, and one of the least disruptive in terms of loss of cleavage and polyadenylation efficiency [16]. Multiple potential polyadenylation motifs within a single gene are increasingly recognised to play a role in post-transcriptional regulation of gene expression. In a comprehensive screen of UTR length, Mangone *et al.* have recently shown that in C elegans, approximately 50% of genes have >1 polyadenylation site, giving rise to variable length UTRs [20]. Mangone *et al.* identified developmental stage-specific isoforms of many transcripts, with a preponderance of longer isoforms in earlier developmental stages [20]. The relatively high diversity in polyadenylation sites employed in lower organisms such as C elegans suggests that polyadenylation site recognition may be more flexible than in higher eukaryotes [20]. However, there is also robust evidence for coordinated shortening of UTR length in higher eukaryotes. Proliferating murine CD4+ T cells exhibit increased expression of mRNAs terminating at upstream polyadenylation sites [21]. Tumor-derived cell lines express substantial amounts of mRNA isoforms with shorter 3′UTRs [22]. In both cases, UTR shortening enables relief of post-transcriptional repression by microRNAs (miRs), and leads to increased expression of the proteins in question. In the current study, we found that the short isoform of TGF-β_1_ predominated in a wide variety of normal human tissues and cells. However, this does not exclude the longer isoform playing an important role in disease states, or earlier developmental stages or organisms.

The above data shows that while the long form of TGF-β_1_ 3′UTR is highly inhibitory to translation, it is expressed at low levels in adult human tissues, and is suggestive that TGF-β_1_ transcripts with the short 3′UTR play a dominant role in TGF-β_1_ synthesis in adult human tissues, and in cells of diverse lineage. The data also shows that the short 3′UTR is inherently inhibitory to protein synthesis, suggesting the potential for post-transcriptional regulation of this transcript by miRs. Use of multiple predictive algorithms suggested targeting of the short UTR by miR-744. Subsequently, miR-744 was confirmed experimentally to repress TGF-β_1_ synthesis, and direct targeting of TGF-β_1_ was established using a 3′UTR reporter construct.

miR-744 has been identified in a broad range of cells and tissues in sequencing-based screens of small RNA libraries [23], [24]. Increased expression of miR-744 has been detected in B lymphocyte-derived cell lines and peripheral blood mononuclear cells from patients with lupus nephritis [25]. In the current study, we have shown widespread expression of miR-744 in human tissues. It will be interesting in future studies to characterise the role of miR-744 in the regulation of TGF-β_1_ in pathological contexts characterised by increased TGF-β_1_ synthesis.

TGF-β_1_-dependent changes in miR expression are important effectors of cellular responses. Our recent data demonstrate that TGF-β_1_-mediated suppression of miR-192 facilitates epithelial to mesenchymal transition in renal epithelial cells [26], while Thum *et al.* have shown that increased expression of miR21 downstream of TGF-β_1_ leads to cardiac fibrosis [27]. miRs are also known to regulate TGF-β_1_ signalling, including repression of Smad2 by miR155 [28], and targeting of multiple components of the TGF beta-Smad signalling pathway by the miR17-92 cluster [29]. In the current study, we have shown that miRs also target TGF-β_1_ directly, further emphasising the important role that miRs play in TGF-β_1_-directed cellular responses, and demonstrating a novel mechanism by which TGF-β_1_ synthesis may be controlled.

## Materials and Methods

### General laboratory reagents

Except as otherwise stated, reagents were purchased from Sigma-Aldrich (Poole, UK), Promega (Southampton, UK), New England Biolabs (MA, USA), and Invitrogen (Paisley, UK) unless stated otherwise. Oligonucleotides were purchased from ThermoFisher Scientific (MA, USA).

### Cell culture

HK-2 cells, a clonal E6/E7-transformed human renal proximal tubule epithelial cell line [30], were maintained in DMEM/Ham's F-12 medium supplemented with 10%FCS (Biological Industries Ltd, Cumbernauld, UK) 2 mM L glutamine, 5 mg/ml insulin, 5 mg/ml transferrin, 5 ng/ml sodium selenite and 0.4 mg/ml hydrocortisone, at 37°C in a 5% CO_2_ humidified incubator. The growth medium was replaced every 3–5 days. Transfection was typically carried out on cells that were 80% confluent.

### RNA extraction

RNA was extracted from cells in culture using TriReagent according to the manufacturers protocol. The RNA was resuspended in 20 µl of water and 1 µg was reverse transcribed by the High Capacity cDNA Reverse Transcription (RT) kit (Applied Biosystems, CA, USA)).

### Polymerase Chain Reaction

PCR for the two forms of 3′UTR was carried out using the following primers which incorporate the Xba1 restriction site to facilitate cloning:

Forward CCTCTAGAGGTCCCGCCCCGCCCCGCCCC

Reverse short form of 3′UTR CCTCTAGACCTCTCTCCATCTTTAATGGGG

Reverse long form of 3′UTR CCTCTAGACAGGCGTGAGCCACCCCGCCTGGCCT

PCR reactions contained 0.25 µl DNA polymerase (AGS Gold, 5 U/µl, Hybaid, Essex, UK) 5 µl of 10× PCR buffer (AGS, Hybaid), 3 µl of d-nucleotides triphosphate (dNTP) (10 µM), 1.25 µl of sense primer (20 µM), 1.25 µl of anti-sense primer (20 µM), 2 µl of cDNA template, (equivalent to 0.2 µg of the RNA transcribed), 2 µl of DMSO, and deionized water to make a final volume of 50 µl. The reaction consisted of a DNA denaturing step of 94°C for 4 min, followed by 35 cycles of a denaturing step for 30 s at 94°C, primer annealing for 30 sec at 55°C, and extension for 60 sec at 68°C. The PCR was concluded with a final extension for 15 min at 68°C. Negative RT controls show no bands on the gel following PCR, confirming that the products seen were not due to genomic contamination.

### Plasmid construction

The PCR products were separated on a 2% agarose gel, purified and cut with Xba1 and then ligated into a pGL3-control vector (Promega) that had been linearised by cutting at the Xba site which is located 3′ to the luciferase coding region. The resulting constructs, pGL3Long and pGL3short, were then sequenced to ensure complete homology with the published sequences and correct orientation in the vector.

### Luciferase Reporter System

The dual reporter assay system (Promega) was used to examine the effects of the different 3′UTR sequence lengths on the luciferase activity relative to that of Renilla in this reporter assay system. Transfection was carried out using Lipofectamine LTX and the Plus reagent (Invitrogen) as recommended. Cells were growth arrested in the absence of serum for 4 h prior to transfection with the luciferase reporter constructs (pGL3-control) together with the Renilla luciferase control plasmid (pRL-SV40) at a ratio of 9∶1. 24 h following transfection the cells were lysed in passive lysis buffer. The firefly and renilla luciferase activity were determined by the dual luciferase reporter assay kit (Promega) and luciferase measured on a Fluostar Optima luminometer.

### miR transfection

The transfection of miRs into HK2 cells was carried out at a final concentration of 50 nM of miR. In co-transfection experiments, the amount of plasmid luciferase construct used was reduced from 0.2 µg/well of a 24 well plate to 0.05 µg/well as preliminary experiments suggested that this was the optimum ratio of plasmid DNA to miR oligonucleotide. Scrambled miR and miR-16 were transfected in parallel to the miRs of interest as negative and irrelevant controls. Successful transfection of HK-2 cells with the miRs of interest was confirmed by RT-qPCR.

### RT-qPCR for Luciferase mRNA

RNA was extracted 24 h following transfection of 6-well plates using a total RNA isolation kit (Agilent Technologies, Wilmington, USA). The samples were DNAse I treated to remove plasmid DNA contamination. cDNA was generated as described previously. Primers to luciferase and GAPDH mRNA were designed using Primer3 (http://frodo.wi.mit.edu/primer3/input.htm)

GAPDH-forward CCTCTGACTTCAACAGCGACAC

GAPDH-reverse TGTCATACCAGGAAATGAGCTTGA,

luciferase-forward GGTCCTATGATTATGTCCGGTTATGT

luciferase-reverse CGTCTTCGTCCCAGTAAGCTATGT.

The mRNA was quantified by RT-qPCR according to standard protocol using “power sybr® green PCR Master Mix” (Applied Biosystems) on a 7900HT Fast Real-Time PCR System (Applied Biosystems). The relative changes in gene expression were analysed by the 2 ^−ΔΔCT^ method.

### Quantification of total TGFβ1 protein by ELISA

HK-2 cells were growth arrested prior to transfection in serum free medium as described above. Parallel wells were treated with transfection reagent alone, or with control miR oligonucleotides. The cells were incubated for up to 72–96 h, at which time the medium was harvested and the total TGF*β1* was quantified by sandwich enzyme – linked immunosorbant assay (R and D Systems, Minneapolis, USA) as described previously [8].

### Quantification of TGFβ1 mRNA and 3′UTRs by RT-qPCR

In order to determine the copy number of each form of UTR, a reference plasmid was generated incorporating TGF-β_1_ sequence from the end of the last intron junction to the 3′ end of the reference sequence (Genebank NM_000660.3). Absolute quantification was performed including log10 dilutions of reference standard. Long- and total- TGFβ UTR sequence were assayed using the PCR primers:

TGFB1 Total F

GCCCTGTACAACCAGCATAAC

TGFB1 Total R

CACGTAGTACACGATGGGCA

TGFB1 Long F

ACTGCGGATCTCTGTGTCATTG

TGFB1 Long R

CAGTAGTGTTCCCCACTGGTC

### Quantification of microRNA by RT-qPCR

The presence and relative amounts of miR-744 and miR-16 as endogenous control were determined by qPCR using Applied Biosystems TaqMan MicroRNA Assays [Product IDs 002324 and 000391] (Applied Biosystems, Lingley House, 120 Birchwood Boulevard, Warrington. WA3 7QH UK) as per the manufacturers instructions.

### Statistics

Replicate samples were analysed a minimum of three times and the results were compared using the Students' T Test. P values of 0.05 and under were taken to be significant, and the significant p values are indicated with an asterix in the figure, with the degree of significance given in the corresponding figure legend.
